# Characterization of *Glomerella* Strains Recovered from Anthracnose Lesions on Common Bean Plants in Brazil

**DOI:** 10.1371/journal.pone.0090910

**Published:** 2014-03-14

**Authors:** Quélen L. Barcelos, Joyce M. A. Pinto, Lisa J. Vaillancourt, Elaine A. Souza

**Affiliations:** 1 Departamento de Biologia, Universidade Federal de Lavras, Lavras, Minas Gerais, Brazil; 2 Empresa Brasileira de Pesquisa Agropecuária (Embrapa), Sinop, Mato Grosso, Brazil; 3 Department of Plant Pathology, University of Kentucky, Lexington, Kentucky, United States of America; State Key Laboratory of Pathogen and Biosecurity, Beijing Institute of Microbiology and Epidemiology, China

## Abstract

Anthracnose caused by *Colletotrichum lindemuthianum* is an important disease of common bean, resulting in major economic losses worldwide. Genetic diversity of the *C. lindemuthianum* population contributes to its ability to adapt rapidly to new sources of host resistance. The origin of this diversity is unknown, but sexual recombination, via the *Glomerella* teleomorph, is one possibility. This study tested the hypothesis that *Glomerella* strains that are frequently recovered from bean anthracnose lesions represent the teleomorph of *C. lindemuthianum*. A large collection of *Glomerella* isolates could be separated into two groups based on phylogenetic analysis, morphology, and pathogenicity to beans. Both groups were unrelated to *C. lindemuthianum*. One group clustered with the *C. gloeosporioides* species complex and produced mild symptoms on bean tissues. The other group, which belonged to a clade that included the cucurbit anthracnose pathogen *C. magna*, caused no symptoms. Individual ascospores recovered from *Glomerella* perithecia gave rise to either fertile (perithecial) or infertile (conidial) colonies. Some pairings of perithecial and conidial strains resulted in induced homothallism in the conidial partner, while others led to apparent heterothallic matings. Pairings involving two perithecial, or two conidial, colonies produced neither outcome. Conidia efficiently formed conidial anastomosis tubes (CATs), but ascospores never formed CATs. The *Glomerella* strains formed appressoria and hyphae on the plant surface, but did not penetrate or form infection structures within the tissues. Their behavior was similar whether the beans were susceptible or resistant to anthracnose. These same *Glomerella* strains produced thick intracellular hyphae, and eventually acervuli, if host cell death was induced. When *Glomerella* was co-inoculated with *C. lindemuthianum*, it readily invaded anthracnose lesions. Thus, the hypothesis was not supported: *Glomerella* strains from anthracnose lesions do not represent the teleomorphic phase of *C. lindemuthianum*, and instead appear to be bean epiphytes that opportunistically invade and sporulate in the lesions.

## Introduction

Anthracnose, caused by the fungus *Colletotrichum lindemuthianum* (Sacc. & Magn.) Scribn., is one of the most important diseases on common bean (*Phaseolus vulgaris* L.) worldwide, and causes significant losses in Brazil [1], [2]. Anthracnose is generally managed by the use of resistant cultivars, but the extreme genetic diversity of the pathogen population in Brazil contributes to frequent failure of resistance sources [3]–[8]. It is unclear how diversity arises, but genetic recombination during sexual reproduction is one possibility. The teleomorph of *C. lindemuthianum*, known as *Glomerella lindemuthiana* Shear (syn. *G. lindemuthianum, G. cingulata* (Stonem.) Spauld. et Schrenk f. sp. *phaseoli*), was first described in 1913 by Shear and Wood [9]. Mating in *Glomerella* has been studied in several species, including *G. lindemuthiana*, in culture. All reports agree that, although some strains of *Glomerella* can be homothallic, the majority of fertile strains are heterothallic [10]–[16]. All reports also agree that mating in *Glomerella* is not regulated by a single mating type locus, and that there are a large number of compatible mating types in the population, which could indicate a large potential for outcrossing [16]. Studies of a highly fertile population of *G. cingulata* strains from morning glory in the early part of the 20^th^ century suggested that the common occurrence of asexual strains was due to frequent mutations in fertility genes, and that multiple mating compatibilities were the result of complementation among these mutations, the so-called “unbalanced heterothallism” theory [17]–[28]. Previous reports suggest that sexual fertility is rare in *G. lindemuthiana* [29]–[31]. Sexual recombination has been demonstrated among a handful of fertile strains in the lab, but it has not been shown to occur in the field. Thus the contribution of sexual recombination to pathogen population diversity in *C. lindemuthianum* remains unclear.

We have isolated a large number of *Colletotrichum* strains from anthracnose lesions on naturally infected common beans. Some single-spored strains produced the *Glomerella* teleomorph readily when cultured alone, while others appeared to be asexual in culture [11], [13], [32], [33]. *Glomerella* strains isolated from bean anthracnose lesions in Brazil have usually been identified as *G. cingulata* f. sp. *phaseoli* or *G. lindemuthiana*, under the assumption that they represent the teleomorphic phase of *C. lindemuthianum* [11], [13], [32], [34]. However, we recently reported that several of these teleomorphic strains produced no, or only mild symptoms, when reinoculated onto beans susceptible to anthracnose [5], [11], [13], [32]. We have further reported that some sexual and asexual strains can be distinguished by morphology [35], and by molecular fingerprint [5]. Our goal in this study was to characterize the diversity and the pathogenic and sexual behavior among a larger population of these teleomorphic strains, and to determine their relationship with asexual *C. lindemuthianum* strains that cause bean anthracnose. The work described here reveals that *Glomerella* isolates from bean anthracnose lesions belong to two different genetic lineages, neither of which is closely related to *C. lindemuthianum*. Members of one lineage did not cause symptoms when inoculated on bean tissues, while those from the other lineage caused only very mild symptoms. Our conclusion is that the *Glomerella* strains are epiphytes that opportunistically colonize the anthracnose lesions produced by *C. lindemuthianum*.

## Materials and Methods

### Isolates and Culture Conditions

*Colletotrichum* isolates were collected from anthracnose lesions on pods, leaves and petioles of common bean (*P. vulgaris*) from naturally infected fields. The collections were made primarily in the cities of Lavras, Lambari, and Ribeirão Vermelho, in the southern state of Minas Gerais (Table S1). No specific permissions were required for collections in these locations and studies did not involved endangered or protected species. The study was conducted in the Department of Biology of the Universidade Federal of Lavras, Minas Gerais, Brazil and in the Plant Pathology Department of the University of Kentucky, Kentucky, United States. Small pieces of infected tissue were disinfected and deposited in Petri dishes containing M3 culture medium [36]. A total of 68 single-spored isolates, recovered from 68 individual lesions, were fertile and produced the *Glomerella* teleomorph in culture. One perithecium each from 54 of these isolates was crushed, and a strain derived from a single ascospore was recovered for each isolate. For the remaining 14 isolates (UFLAG05, UFLAG06, UFLAG07, UFLAG08, UFLAG15, UFLAG21, UFLAG30, UFLAG43, UFLAG47, UFLAG54, UFLAG73, UFLAG104, UFLAG106, and UFLAG112), between two and five monoascospore strains were recovered from a single crushed perithecium of each (Table S1). All monoascospore strains were maintained on M3 media at 22°C in the dark.

### Morphological Characterization

#### Colony classification

**Figure 1:**
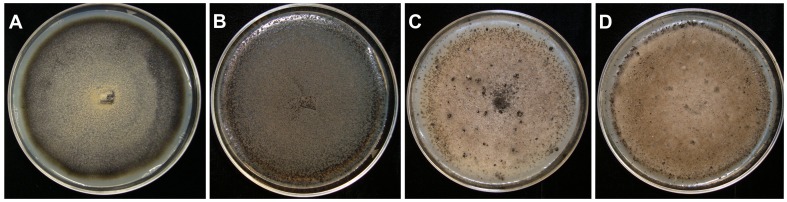
Morphology of colonies recovered after isolating single ascospores from *Glomerella* sp. strains. A) Black conidial A colony, producing masses of conidia; B) Black conidial B colony, with sparse production of conidia; C) White Plus colony, producing clumps of perithecia; and D) White Minus colony, producing scattered perithecia.

The monoascospore strains were classified into four different morphological classes: 1) Conidial A strains did not produce perithecia on M3 media, but produced abundant conidia in large masses; 2) Conidial B strains did not produce perithecia, and produced conidia sparsely, scattered over the colony surface; 3) Plus strains formed perithecia in clumps; 4) Minus strains produced individual perithecia scattered across the surface of the culture (Figure 1 and Table S1). A few of the plus and minus strains also produced conidia, but production was sparse. Thus, it was not possible to collect and evaluate both ascospores and conidia from a single strain. Henceforth, the Conidial A and B strains will be referred to as “conidial” strains, and the Plus and Minus strains as “perithecial” strains. A total of 88 monoascospore strains, comprising all four classes, and including one or more representatives of 61 of the 68 original *Glomerella* single-spored isolates, were used in the experiments described below (Table S1).

#### Index of mycelial growth rate (IMGR) and colony diameter

The experiment was a completely randomized design (CRD) with four replicates. Each plot consisted of a single Petri dish (80 mm diameter). Mycelium plugs 6 mm in diameter were placed in the centers of individual Petri dishes containing M3 media, and cultures were incubated in the dark at 22°C. Colony diameter (millimeters) was measured twice at right angles for each colony at intervals of 24 hours over the course of 8 days, and the averages were used to estimate the IMGR according to the expression 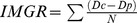, where Dc is the current average of the colony diameter, Dp is the average of the previous colony diameter, and N is the number of days after inoculation [37]. Final colony diameter (mm) was determined after 8 days of incubation.

#### Germination rate

The experiment was a completely randomized design (CRD), split plot in time, with two replicates. Ascospore and conidial suspensions were adjusted to 1.2×10^6^ spores/mL, and 500 µl of each suspension was spread in Petri dishes containing water agar (2%). After 24 and 48 hours of incubation at 22°C in the dark, 50 spores per replicate were observed by light microscopy with an Olympus CX41 (Olympus Deutschland GmbH, Hamburg, Germany). Spores that had produced germ tubes with a length equal to or greater than the smallest diameter of the spore were considered to be germinated.

#### Spore measurements, septum formation and Conidial Anastomosis Tubes (CATs)

For all experiments, 200 µl of a spore suspension (1.2×10^6^ spores/ml) of each strain was applied to a chambered borosilicate coverglass (Nalge Nunc International, Rochester, NY) and incubated at 22°C in the dark. Samples were examined by using an inverted epifluorescence microscope (Zeiss Axio Observer Z1; Carl Zeiss Inc., Jena, Germany) after adding the dye Calcofluor White (Sigma-Aldrich, St. Louis) at a concentration of 0.12 M. The fluorescence was detected at 420/70 nm using the 40× objective. The images were captured using Zeiss Axiovision software and processed using ImageJ 1.41 software (U.S. National Institutes of Health, Bethesda, MD).

Measurements were made for 30 non-germinated spores of each strain in a CDR experiment. The width and length of the ungerminated spores (µm) were measured by using the imaging software Image Tool 3.0 (University of Texas Health Science Center, San Antonio).

The presence or absence of a septum was observed after 24 hours in two replicates of 100 germinating spores. The formation of CATs was quantified 24 hours after incubation as the percentage of spores involved in anastomosis [33]. Two replicates consisting of 200 spores each were analyzed.

#### Statistical analyses

Morphological data were subjected to analysis of variance (ANOVA), and means were compared by the Scott-Knott test (P = 0.05), by using the statistical program MSTAT-C 1.0 (Michigan State University, East Lansing) and SISVAR [38], respectively.

#### Sexual interactions

The 73 monoascospore strains were paired on M3 media in all possible combinations (2701 pairings) and incubated at 22°C in the dark. Mycelial plugs 6 mm in diameter were placed at a distance of 2 cm apart, with two repetitions per confrontation. Confrontations were evaluated after 15 days of incubation.

The formation of a line of perithecia containing ascospores at the contact zone of paired strains was an indication of a fertile interaction between the strains. To further analyze fertile interactions, dialysis membranes were used to separate the partners. Dialysis membranes do not allow physical contact between strains but permit the exchange of molecules that can induce production of fertile selfed perithecia in some strains of *Glomerella* [26]. Fertile interactions were classified as: a) induced homothallic, when a line of perithecia was formed even when the partners were separated by a dialysis membrane; or b) likely heterothallic, when a line of perithecia was produced, but only in the absence of a dialysis membrane.

### Molecular Characterization

#### DNA extraction, PCR reactions and sequencing

Mycelium plugs were transferred to 125 mL of M3 liquid medium in Erlenmeyer flasks. The flasks were shaken at 110 rpm/min at 22°C for 7 days in the dark. Mycelium was dried in a vacuum, and subsequently freeze-dried for 48 hours. DNA was extracted using a high-throughput DNA prep method [39], or a mini-prep method [40].

Sequences of the internal transcribed spacer (ITS variable regions) of the ribosomal DNA and the high mobility group (HMG)-encoding sequence of the MAT1-2-1 mating type gene were amplified by using polymerase chain reaction (PCR). The universal primers ITS1 and ITS4 were used for amplification of ITS variable regions [41]. PCR reactions contained 10–100 ng of genomic DNA, 2.5 mM MgCl_2_, 1X PCR buffer, 0.2 mM each dNTP, 2.5 U of Taq DNA polymerase (Invitrogen, Carlsbad, California) and 0.5 µM of each primer. The amplification cycle consisted of denaturation at 95°C for 5 min followed by 40 cycles consisting of 30 s at 95°C, 30 s at 50°C and 1.5 min at 72°C.

To amplify HMG sequences, primers HMGCLF and HMGCLR were used. This primer pair is specific to the *C. lindemuthianum* MAT1-2-1 HMG region [10]. The amplification cycle consisted of denaturation at 95°C for 5 min followed by 40 cycles consisting of 30 s at 95°C, 30 s at 50°C, and 1.5 min at 72°C. The degenerate primers NcHMG1 and NcHMG2, and PCR conditions described previously [42], were used to amplify HMG sequences from strains that were not amplified by the HMGCLF primer pair. PCR products were cloned in pGEMTeasy (Promega Corporation, Madison, USA) and sequenced with primers complementary to the cloning vector. A new primer pair, HMGglo1 (5′CGAGCTCCGTGTATCTCTGG3′) and HMGglo2 (5′AAAGATCACTGCGCCAA GTT3′), was developed based on these sequences. PCR reactions contained 10–100 ng of genomic DNA, 2.5 mM MgCl_2_, 1X PCR buffer, 0.2 mM each dNTP, 2.5 U of Taq DNA polymerase (Invitrogen, Carlsbad, California) and 0.2 µM of each in 50 µL. The amplification cycle consisted of denaturation at 95°C for 5 min followed by 40 cycles consisting of 30 s at 94°C, 30 s at 55°C and 30 s at 72°C. PCR products were separated in a 1% agarose gel, stained with ethidium bromide, and viewed on a UV transilluminator.

HMG amplicons of 10 strains, and ITS amplicons of 17 strains, were recovered from the electrophoresis gels with a gel extraction system (QIAGEN Inc., Valencia, California) (Table S1). These PCR products were sequenced with the BigDye terminator cycle sequencing kit (Applied Biosystems, Foster City, California), and the sequences were analyzed on an ABI 310 automated sequencer (Applied Biosystems).

#### Fungal transformation

To obtain strains expressing the green fluorescent protein (GFP), an *Agrobacterium tumefaciens*-mediated transformation protocol was used [43]. The strains LV115 and UFLAG06 were transformed (Table S1). The vector used was pJF1 [43], containing the SGFP gene driven by the TOX-A promoter from *Pyrenophora tritici-repentis* [44], and with the hygromycin phosphotransferase gene as selectable marker. The transformants were selected on PDA media containing 50 µg/ml of hygromycin B, and purified by single sporing.

#### Phylogenetic analysis

Phylogenetic analyses were performed by using the Phylogeny.fr platform with default settings [45], and included the following steps: sequence alignment with MUSCLE (v3.7); removal of ambiguous regions with Gblocks (v0.91b); construction of a phylogenetic tree using the maximum likelihood method with PhyML (v3.0 aLRT); and evaluation of internal branch reliability using the aLRT test (SH-Like). Phylogenetic trees were drawn and edited using TreeDyn (v198.3). Trees were drawn with midpoint rooting, and all branches with values of less than 0.50 were collapsed. The ITS sequence alignment is provided in File S1.

### Infection Assays

#### Spore solutions and inoculations

Sterile bean pods were inoculated with each strain and incubated at 22°C for 10 days in the dark. Only six of the strains sporulated on the pods (Table S1). For the other strains, six plates containing M3 culture medium were inoculated and incubated at 22°C for 10 days in the dark to produce spores. Spore solutions were made by harvesting and washing three times in sterile water with centrifugation and resuspension in water. The concentration was adjusted to 1.2×10^6^ spores/mL for all experiments. Inoculations were made by spraying the spore solutions on plant seedlings to runoff. Alternatively, 5 µL drops were placed on detached hypocotyls or leaves in humidity chambers.

#### Pathogenicity tests

Pathogenicity tests were carried out with the 53 monoascospore strains that produced conidia on either M3 medium or bean pods (Table S1). The strains were inoculated on a differential set of 12 cultivars of common bean [46]. The susceptible cultivar Pérola was used as a control. The seeds were sown in trays filled with the growth substrate Multiplant (Terra do Paraíso Ltda., Brazil). Conidial suspensions were sprayed to runoff on ten-day-old seedlings. The inoculated bean seedlings were incubated in a fog chamber at 22°C, with a photoperiod of 12 hours, and a relative humidity of 95%, for 48 hours. The trays were then transferred to the greenhouse, and the seedlings were evaluated for severity of anthracnose ten days after inoculation using the diagrammatic scale [47]. Seedlings with ratings between 1 and 3 were considered resistant, and those with ratings greater than 3 were considered susceptible. The identification of races was made as proposed by Habgood [48].

Detached hypocotyls of the Pérola and Michelite cultivars were inoculated in order to directly observe the development of the fungi in the bean tissues. For these infection assays, five strains were used (Table S1). They included one known pathogenic, asexual *C. lindemuthianum* strain (LV115) as a control [32]. UGFLAG06-1 (Plus strain, ascospores were used for inoculation), and UFLAG06-2 (Conidial B strain, conidia were used for inoculation), were both derived from a single perithecium of the monoascsopore strain UFLAG06 (Table S1). Strains UFLAG08-2 (Minus strain, ascospores were used for inoculation), and UFLAG08-3 (Conidial A strain, conidia were used for inoculation), were both derived from a single perithecium of the monoascospore strain UFLAG08 (Table S1). UFLAG06 and UFLAG08 were derived from different fertile single-spored isolates collected from two different lesions on naturally infected beans (Table S1). Detached hypocotyls from ten-day-old seedlings were placed into sterile Petri dishes lined with moistened germination paper. The Petri dishes were placed into a germination box, and the hypocotyls were inoculated with 5 µl drops of spore solutions and incubated at 22°C for seven days. The control was inoculated with drops of sterile water. Symptoms were observed and photographed 10 days after inoculation.

#### Light microscopy experiments

Inoculations using conidia of *C. lindemuthianum* strain LV115 and ascospores of strain UFLAG06 (Plus strain), were made on detached leaves and hypocotyls of the susceptible common bean cultivar Pérola, and the resistant cultivar G2333. Observations were made at 24, 48, 72, 96, and 120 hours after inoculation (hpi). Samples were obtained by hand-sectioning the plant tissue with a razor blade. To clarify them, the plant tissue samples were immersed in a solution of methanol: chloroform: acetic acid (60∶30:10) for 30 minutes. Samples were then immersed in Trypan Blue (250 µg/ml) in lactophenol solution (lactic acid: phenol: H_2_0 1: 1∶1) for 20 minutes. Samples were transferred to lactophenol for 30 min, and then observed in 50% glycerol by using a Zeiss Axioscop. Images were obtained with AxioVision 4.8 software (Carl Zeiss, Oberkochen, Germany).

At 72 hpi, the percentage of spores forming appressoria was measured in a completely randomized design (CRD) experiment, split plot in time, with three replicates. Each plot consisted of a sample of 100 spores. ANOVA was conducted using MSTATC 1.0 (Michigan State University, East Lansing).

#### Fluorescence microscopy

The transformed fertile monoascospore strain tQB01, and the transformed *C. lindemuthianum* strain tQB02, both expressing GFP, were inoculated on detached hypocotyls and leaves of the susceptible cultivar Pérola and the resistant cultivar G2333. Observations were made at 72, 96 and 120 hpi.

To cause localized cell death on the plant tissues, small pieces of dry ice were placed on hypocotyls and on leaves of susceptible and resistant cultivars for three seconds. The fertile tQB01 strain was inoculated onto the killed tissues immediately after treatment. Observations were made at 24, 48 and 72 hpi. Inoculated plant tissues were sectioned and observed in 50% glycerol by using a Zeiss Axioscop equipped with epifluorescence and a GFP filter. Images were obtained by using the AxioVision 4.8 software (Carl Zeiss, Oberkochen, Germany).

#### Co-inoculations

Wild type and transformed GFP-expressing strains were co-inoculated on detached hypocotyls of the susceptible cultivar Pérola. The co-inoculations were made in two combinations: 1) Non-fertile *C. lindemuthianum* wild type strain LV115 and fertile transformed monoascospore strain tQB01; and 2) Fertile wild-type monoascospore strain UFLAG06 and *C. lindemuthianum* transformant strain tQB02. The wild type of each strain inoculated alone on Pérola hypocotyls, and mock inoculations with sterile water, were the controls for this experiment. Spore suspensions of each strain were prepared, combined, and then 5 µL drops of the combined suspensions were placed on the plant tissue. Evaluations were made at 120 hpi. Sections of plant tissue were observed in 50% glycerol by using a Zeiss Axioscop equipped with epifluorescence and a GFP filter. Images were obtained by using the AxioVision 4.8 software (Carl Zeiss, Oberkochen, Germany).

## Results

### Morphological Characterization

#### Colony classification

The color of the monoascospore strains in culture on M3 medium was black, brown, or white (Figure 1). The strains comprised four distinct groups based on their morphology and the production of spores: Conidial A (24 strains) and conidial B (29 strains) both produced abundant conidia in culture. In the conidial B group the conidia were scattered over the surface of the agar while in conidial A the conidia were formed in clumps. Plus (11 strains) produced perithecia in clusters while in Minus (9 strains) the perithecia were scattered across the surface of the agar. Plus and Minus strains were always white, while conidial A and B strains were either black or brown (Figure 1). More than one type could be recovered from a single perithecium (Table S1). These four groups were reminiscent of descriptions of single-ascospore strains of *G. cingulata*, published by Chilton and Wheeler [23]. Conidial A and B strains (aka “conidial strains”) produced only conidia, while Plus and Minus strains (aka “perithecial strains”) produced primarily ascospores, and few or no conidia. For our studies of morphological variation described below, we conducted an analysis of variance. Sources of variation were partitioned among conidial strains (conidial A and B); among perthecial strains (Plus and Minus); and among conidial vs. perithecial strains. All these sources of variation were statistically significantly different (p<0.05).

#### Index of mycelial growth rate (IMGR) and colony diameter

Conidial strains could be statistically separated into two groups on the basis of IMGR, and three on the basis of colony diameter. There was no relationship between these groups and the A or B phenotypes. IMGR ranged from 8.79 mm/day to 10.39 mm/day, and colony diameter ranged from 67.75 mm to 80.0 mm. Perithecial strains could be statistically separated into two groups on the basis of IMGR and on the basis of colony diameter. There was no relationship between these groups and the Plus or Minus phenotypes. IMGR for perithecial strains ranged from 8.89 to 10.5 mm/day, and colony diameter ranged from 68.00 to 79.75 mm.

#### Percentage of germination

Conidial strains could be statistically separated into six groups at 24 h, and five groups at 48 h, based on the rate of conidial germination. At 24 h, the percentage of conidial germination varied from 7.0 to 89.5%, and at 48 h from 28.0 to 97.5%. Perithecial strains could be separated into three classes based on ascospore germination rate at both 24 h and 48 h. At 24 h, the ascospore germination rate ranged from 27.5 to 88.5%, and at 48 h it ranged from 51.5 to 98.5%. Ascospores from perithecial strains germinated significantly faster, on average, than conidia from conidial strains. None of the classes were related to the A, B, Plus, or Minus phenotypes.

#### Spores measurements, septum formation and Conidial Anatomosis Tubes (CAT’s)

Conidial strains formed seven groups for both spore width and length. Width varied from 2.88 to 5.62 µm, and length varied from 8.46 to 15.85 µm. Perithecial strains could be separated into five groups based on spore width and eight groups based on spore length. Width varied from 6.32 to 9.2 µm, and length varied from 20.5 to 32.78 µm. Ascospores were, on average, significantly larger than conidia.

**Figure 2:**
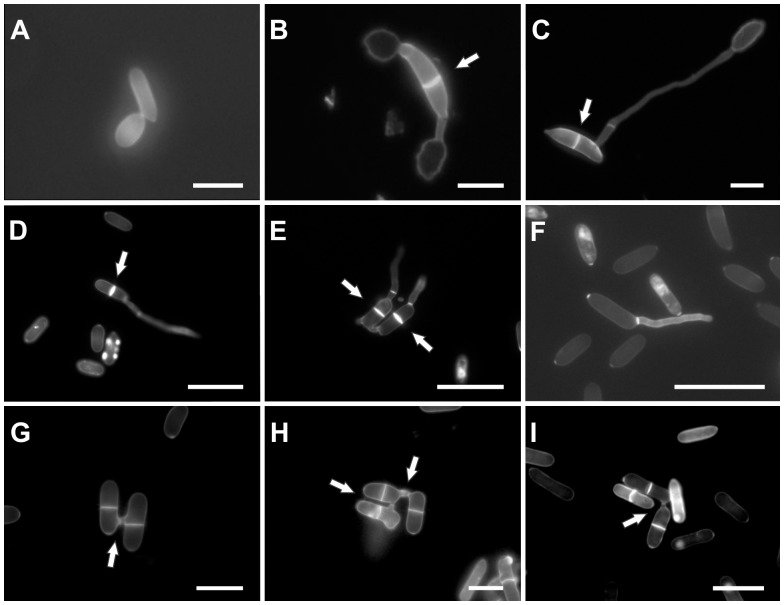
Formation of septa and conidial anastomosis tubes. A) *C. lindemuthianum* strain LV115 did not form a septum during germination; B) *Glomerella* UFLAG08 (Minus strain), germinated ascospore with a septum (white arrow) and two appressoria; C) *Glomerella* UFLAG06 (Plus strain), ascospore with a septum (white arrow) and an appressorium; D) *Glomerella* UFLAG68-1 (conidial A strain), conidia forming a septum (white arrow) during germination; E) *Glomerella* UFLAG36-1 (conidial B strain), two conidia forming septa (white arrows) during germination; F) *Glomerella* UFLAG21-1 (conidial A strain), conidia did not form a septum during germination. Bar: 25 µm. G) CATs formation in the conidial A strain UFLAG07-2. H) CATs formation in the conidial B strain UFLAG117-1; I) CATs formation in the conidial B strain UFLAG111-1. Bar: 20 µm.

Conidia of all conidial strains formed a single septum during germination, with the exception of UFLAG21-1. Ascospores of all perithecial strains also formed a single septum during germination (Figure 2). The percentage of spores in each strain that had formed septa after 24 hours of incubation ranged from 61.5 to 100%.

Conidia from all except two of the 53 conidial strains formed CATs. The 51 strains that formed CATs could be statistically divided into three groups, based on the rate of CATs formation, which varied from 0.75 to 78.75%. The highest percentage of CATs was formed by the conidia of the UFLAG07-2 strain (Figure 2). Ascospores from perithecial strains never formed CATs (not shown).

#### Sexual interactions

**Figure 3:**
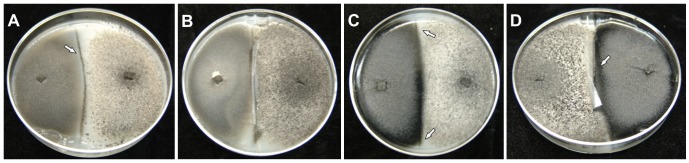
Pairings of monoascospore strains in culture medium with and without dialysis membrane. A) UFLAG47-1 and UFLAG47-2 strains paired without the dialysis membrane, with formation of a contact line with fertile and viable perithecia; B) UFLAG47-1 and UFLAG47-2 strains paired with the dialysis membrane preventing contact between them: the perithecial line is no longer observed; C) UFLAG21-2 and UFLAG104-2 strains paired in the absence of dialysis membrane, with formation of a contact line containing fertile perithecia and viable ascospores; D) UFLAG21-2 and UFLAG104-2 strains paired with the dialysis membrane preventing contact between them: the perithecial line is still observed.

The 73 monoascospore strains were paired in all possible combinations (2701 pairings) on M3 medium. Pairings between conidial A and conidial B strains never resulted in the formation of perithecia (not shown). Pairings between Plus and Minus strains never resulted in the formation of a line of perithecia between the two colonies (not shown). Only 75 pairings (2.78%) resulted in the formation of a line containing perithecia at the point of contact between the strains, and these fertile interactions were only obtained when a conidial strain was paired with a perithecial strain. Twenty-six of these combinations produced only protoperithecia (not shown). The remaining 49 combinations formed fertile perithecia that contained large numbers of asci and ascospores (Table S2). These 49 pairings were repeated, but with a dialysis membrane separating the two strains. Twenty-eight of the combinations no longer formed a contact line of perithecia in the presence of the membrane (Table S2, Figure 3). The other 21 combinations still produced a line of fertile perithecia, even in the absence of physical contact between the strains. In all of these cases, the perithecia were formed on the same side of the membrane as the conidial strain (Figure 3), suggesting that the perithecial strain was inducing its conidial partner to produce fertile, selfed perithecia.

### Molecular Characterization

#### PCR reactions

**Figure 4:**
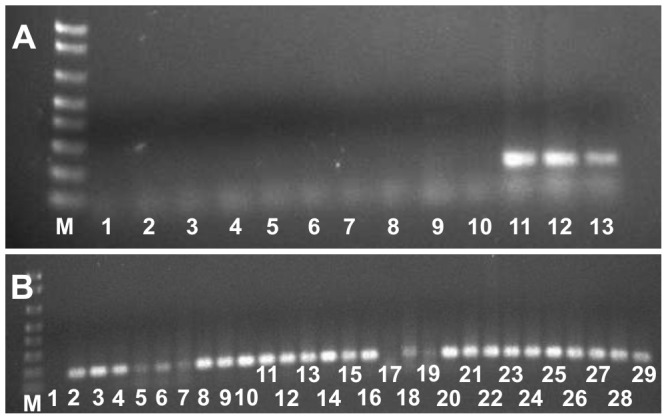
Amplification products of polymerase chain reaction (PCR) with representative conidial and perithecial strains. A) The HMGCLF and HMGCLR primer pair (Garcia-Serrano et al., 2008) only amplified a product from *C. lindemuthianum* strains (lanes 11–13). M) Molecular size marker-100 pb; 1) No template control; 2) Strain UFLAG21-1 (conidial A); 3) Strain UFLAGG21-2 (Plus), 4) Strain UFLAG46-1 (conidial A); 5) Strain UFLAG85-1 (conidial A); 6) Strain UFLAG93-1 (conidial A); 7) Strain UFLAG99-1 (conidial A), 8) Strain UFLAGG110-1 (conidial B); 9) Strain UFLAG112-1 (Minus); 10) Strain UFLAG113-1 (Minus); 11) *C. lindemuthianum* strain LV115; 12) *C. lindemuthianum* strain LV117; 13) *C. lindemuthianum* strain LV120. B) The primer pair HMGgloF and HMGgloR amplified products from the *Glomerella* group 1 strains but the *Glomerella* group 2 strains amplified very poorly (lanes 5–7, and 17–19). M) Molecular size marker-100 pb; 1) No template control; 2) Strain UFLAG21-1 (Conidial A); 3) Strain UFLAG21-2 (Plus); 4) Strain UFLAG23-1 (Conidial B); 5) Strain UFLAG85-1 (Conidial A); 6) Strain UFLAG93-1 (Conidial A); 7) Strain UFLAG99-1 (Conidial A); 8) Strain UFLAG110-1 (Conidial B); 9) Strain UFLAG112-1 (Minus); 10) Strain UFLAG113-1 (Minus); 11) Strain UFLAG43-1 (Minus); 12) Stain UFLAG43-2 (Plus); 13) Strain UFLAG46-1 (Conidial A); 14) Strain UFLAG49-1 (Conidial B); 15) Strain UFLAG73-1 (Plus); 16) Stain UFLAG73-2 (Minus); 17) Strain UFLAG86-1 (Conidial A); 18) UFLAG89-1 (Conidial A); 19) UFLAG92-1 (conidial B); 20) Strain UFLAG15-1 (Plus); 21) Strain UFLAG15-2 (Conidial A); 22) Strain UFLAG54-1 (Conidial A); 23) Strain UFLAG54-2 (Plus); 24) Strain UFLAG68-1 (Conidial A); 25) Strain UFLAG101-1 (Plus); 26) Strain UFLAG104-1 (Minus); 27) Strain UFLAG104-2 (Conidial A); 28) Strain UFLAG117-1 (Conidial B); 29) Strain UFLAG118-1 (Plus).

The ITS1 and ITS4 universal primers successfully amplified a product corresponding in size to the expected ITS product from the 17 perithecial and conidial monoascospore strains that were tested. The primers HMGCLF and HMGCLR [10] amplified a product of the expected size from three control *C. lindemuthianum* strains, but did not amplify a product from any of the 73 monoascospore strains (Figure 4A). However, the specific primers HMGglo1 and HMGglo2 did amplify a single product corresponding in size to the HMG region of the MAT2 locus from most of the monoascospore strains (Figure 4B). The exceptions were the conidial strains UFLAG85-1, UFLAG86-1, UFLAG89-1, UFLAG92-1, UFLAG93-1, and UFLAG99-1, which amplified very poorly with this primer pair (Figure 4B).

#### Phylogenetic analysis

**Figure 5:**
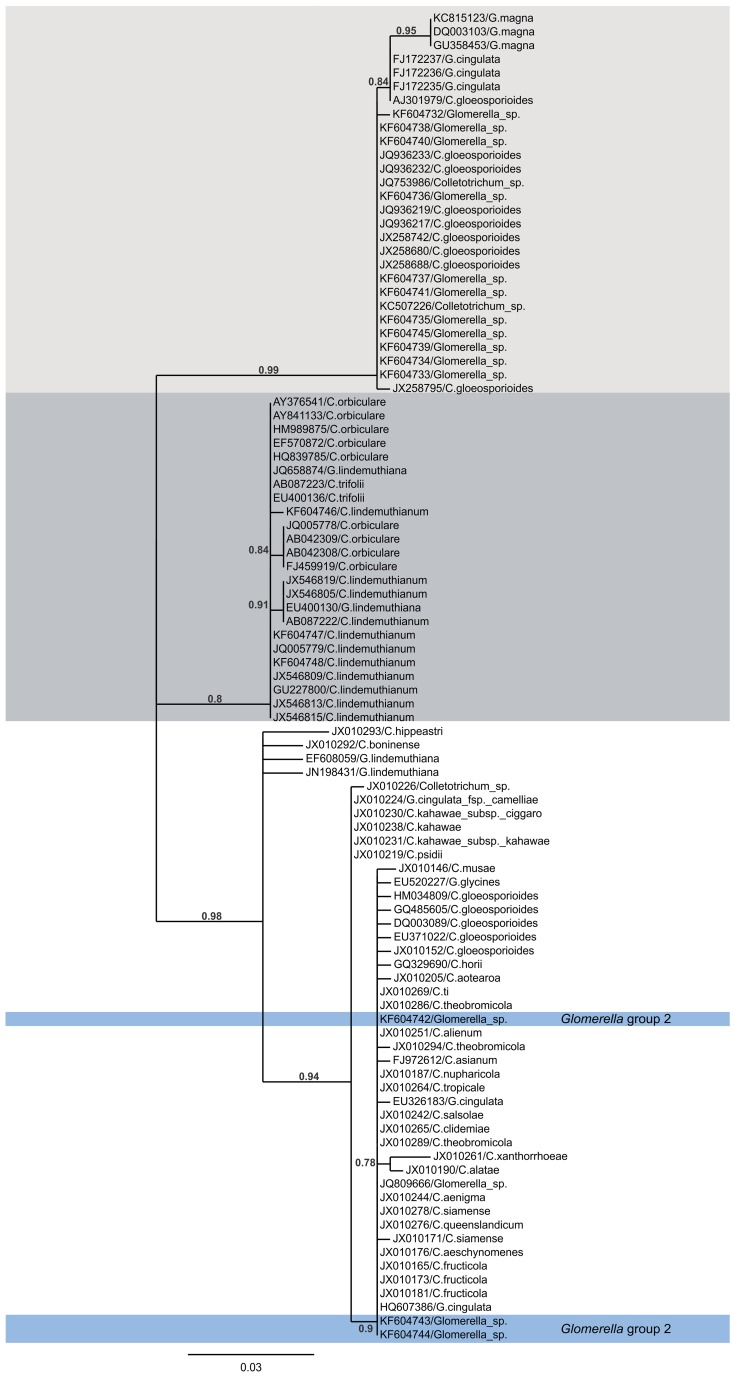
Phylogenetic tree based on ITS sequences of the Brazilian *Glomerella* sp. and *C. lindemuthianum* strains, and other *Colletrotrichum* spp. from Genbank. The tree was constructed by using maximum likelihood analysis, and values above branches indicate the internal branch reliability assessed using the aLRT test (SH-Like) [45]. Scale bar, 0.03 nucleotide replacements per site. Most of the sequenced *Glomerella* sp. monoascospore strains UFLAG02, UFLAG03-1, UFLAG04, UFLAG05-2, UFLAG06, UFLAG06-3, UFLAG07-3, UFLAG08, UFLAG08-4, UFLAG46-1 and UFLAG112 (KF604732, KF604733, KF604734, KF604735, KF604736, KF604737, KF604738, KF604739, KF604740, KF604741 and KF604745, respectively, *Glomerella* group I) clustered together with ITS sequences of *G.magna*, and a few strains identified as *G.cingulata* and *C.gloeosporioides* in Genbank (light gray). Both perithecial and conidial strains were represented. Three Brazilian *C.lindemuthianum* strains LV115, LV117 and LV120 (KF694746, KF604747, and KF604748, respectively) clustered in a separate clade with other sequences from *C. lindemuthianum* and other members of the *C. orbiculare* species aggregate that were available in Genbank (dark gray). ITS sequences of a few of the *Glomerella* sp. monoascospore isolates UFLAG85-1, UFLAG93-1 and UFLAG99-1 (KF604742, KF604743 and KF604744, respectively, *Glomerella* group 2, highlighted in blue) clustered together with sequences from isolates representing the *C. gloeosporioides* species aggregate, including several verified ex-epitype strains [67].

**Figure 6:**
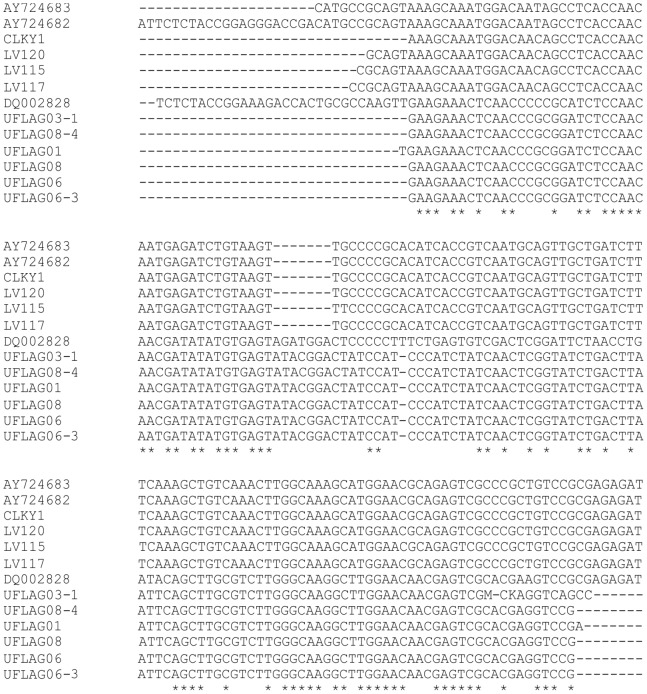
Alignments of HMG sequences. *Glomerella* group 1 (UGLAG01, UFLAG03-1, UFLAG06, UFLAG06-3, UFLAG08 and UFLAG08-4) and *C. lindemuthianum* strains (LV115, LV117 and LV120), aligned with HMG sequences of *C. magna* (DQ002828) and *G. lindemuthiana* (AY724682 and AY724683) from Genbank.

A phylogenetic analysis based on ITS sequences suggested that none of the monoascospore strains are closely related to *C. lindemuthianum* (Figure 5). They also could not be positively identified as another known species of *Colletotrichum* or *Glomerella*. The largest group of isolates clustered with the cucurbit anthracnose pathogen *C. magna* (Figure 5). Other strains in this clade, identified by BLAST homology searches from the Genbank database, were named in the database entries as *C. gloeosporioides* or *G. cingulata*. However, they were distinct from the *C. gloeosporioides* clade that contained sequences of verified ex-epitype strains (Figure 5), and so they had probably been misidentified. The HMG sequences of representatives of this group of monoascospore strains matched only the HMG sequence from *C. magna* in BLAST searches of the Genbank database, and they were distinct from the HMG sequences of *C. lindemuthianum/G. lindemuthiana* (Figure 6). Three of the monoascospore strains (UFLAG85-1, UFLAG93-1, UFLAG99-1) were separate from the larger group, and were located in the ITS phylogeny within the *C. gloeosporioides* species complex (Figure 5).

### Infection Assays

#### Pathogenicity tests

**Figure 7:**
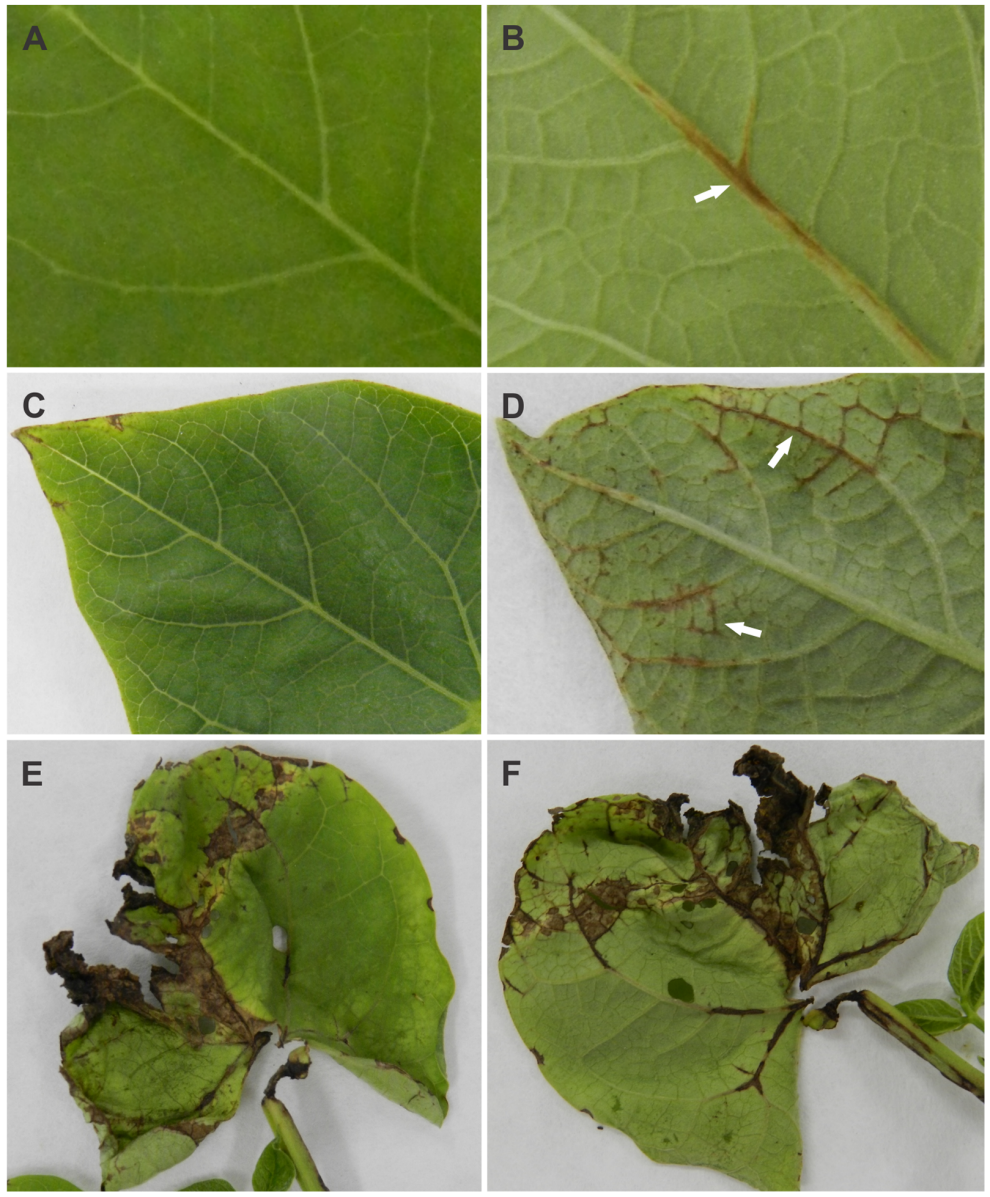
Typical symptoms on Pérola susceptible cultivar (all at 10 dpi). A and B) Leaves inoculated with the conidial strain UFLAG86-1. C and D) Leaves inoculated with the conidial strain UFLAG89-1. E and F) Leaves inoculated with the *C. lindemuthianum* strain LV115.

Of the 53 conidial strains tested, 47 produced no symptoms when conidia were inoculated onto leaves of a set of differential cultivars of common bean, or on the susceptible cultivar that was used as a control (not shown). Only six of the strains (UFLAG85-1, UFLAG86-1, UFLAG89-1, UFLAG92-1, UFLAG93-1 and UFLAG99-1) sporulated well on bean pods, and these same six strains produced mild symptoms on the leaves of the susceptible cultivar, but not on any of the differential cultivars (Figure 7).

**Figure 8:**
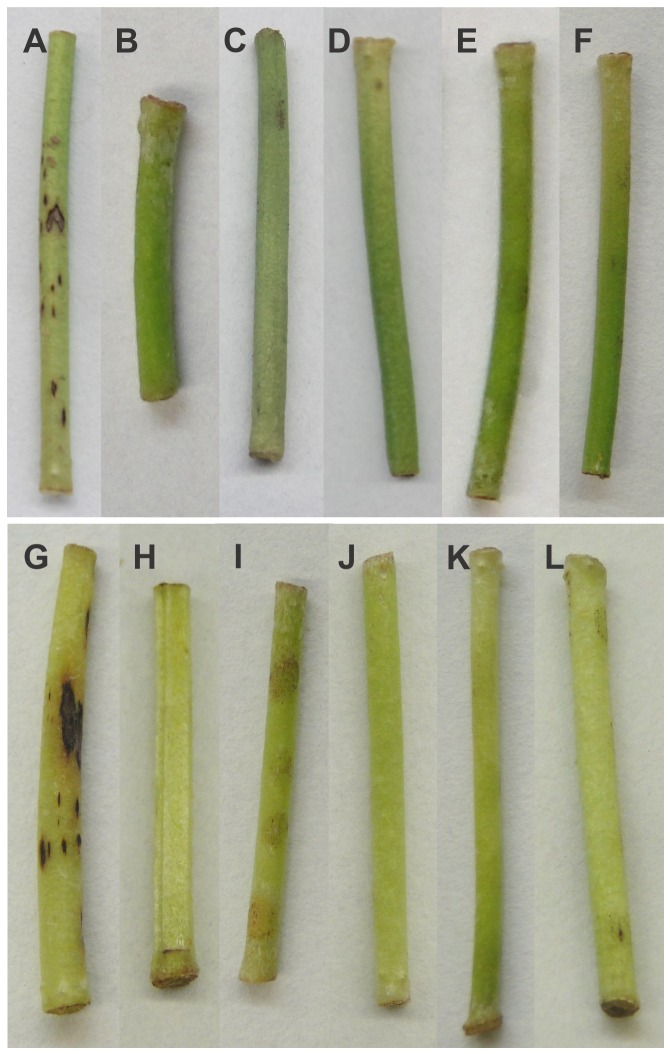
Typical symptoms at 10 A–F) Inoculations on Pérola cultivar; G–L) inoculations on Michelite cultivar; A) Inoculation with conidia of the LV115 *C. lindemuthianum* strain. B) Control mock-inoculated with sterile water; C) Inoculation with ascospores of a perithecial Plus strain (UFLAG06-1); D) Inoculation with conidia of a sibling conidial B strain (UFLAG06-2); E) Inoculation with ascospores of a perithecial Minus strain UFLAG08-2; F) Inoculation with conidia of sibling conidial A strain UFLAG08-3; G) Inoculation with conidia of the LV115 *C. lindemuthianum* strain; H) Control mock-inoculated with sterile water; (I) Inoculation with ascospores of a perithecial Plus strain (UFLAG06-1); J) Inoculation with conidia of a sibling conidial B strain (UFLAG06-2); K) Inoculation with ascospores of a perithecial Minus strain UFLAG08-2; L) Inoculation with conidia of sibling conidial A strain UFLAG08-3.

**Figure 9:**
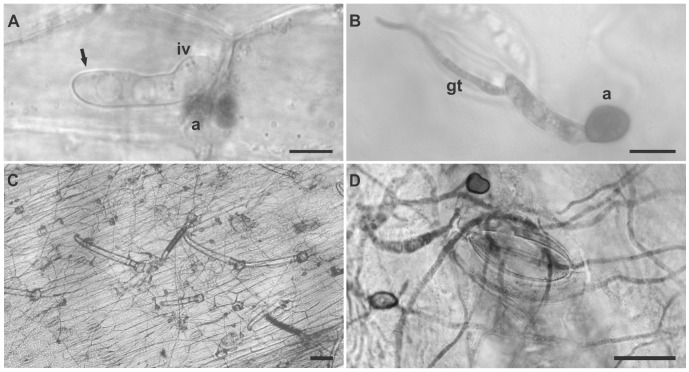
Infection analyses of *C. lindemuthianum* and epiphytic growth of *Glomerella* sp. strain on tissues of the Pérola susceptible cultivar. A) *C. lindemuthianum* strain LV115 on a hypocotyl at 72 hpi, forming appressoria (a), infection vesicle (iv) and primary hyphae (black arrow), Bar: 10 µm. B) *Glomerella* UFLAG06 strain forming appressorium (a) and germ tube (gt) on a leaf at 24 hpi. Bar: 10 µm. C) Epiphytic growth of *Glomerella* sp. strain UFLAG06 on hypocotyl surface of the susceptible cultivar at 24 hpi. Bar: 50 µM. D) Epiphytic growth of *Glomerella* sp. strain UFLAG06 on leaf surface at 48 hpi. Bar: 25 µM.

Inoculation of detached hypocotyls of two susceptible bean cultivars Pérola and Michelite with spores of some of the *Glomerella* strains resulted in no development of anthracnose symptoms. Only conidia of the *C. lindemuthianum* strain LV115 produced symptoms on both susceptible cultivars (Figure 8A and G). For some samples, pale brown discolored spots were seen at the inoculation sites (Figure 8I). Under the light microscope, these were revealed as masses of superficial hyphae and melanized appressoria (Figure 9 C and D). The six strains that produced mild symptoms on leaves (above) were not tested on hypocotyls.

#### Cytology of infection

Typical post penetration intracellular structures, including infection vesicles and primary hyphae, were produced in infected tissues of the susceptible host by the *C. lindemuthianum* LV115 strain by 72 hours after inoculation (hpi) (Figure 9A). Ascospores of the perithecial UFLAG06 strain germinated and produced appressoria within 24 hours (Figure 9B). The *Glomerella* UFLAG06 strain produced abundant appressoria and epiphytic mycelium on common bean tissues at 24 and 48 hpi (Figure 9C and D). By 72 hpi, ascospores of the *Glomerella* strain had produced appressoria at a much higher rate on Pérola (98.33% ±1.52) and G2333 (97% ±3.0) than conidia of the *C. lindemuthianum* LV115 strain (15% ±1.73 and 4% ±1.0, respectively). However, UFLAG06 inoculations were never observed to produce intracellular infection structures, even at 120 hpi. All the sources of variation in the ANOVA were significant (P<0,001).

#### Infection process observed with strains expressing the green fluorescent protein

**Figure 10:**
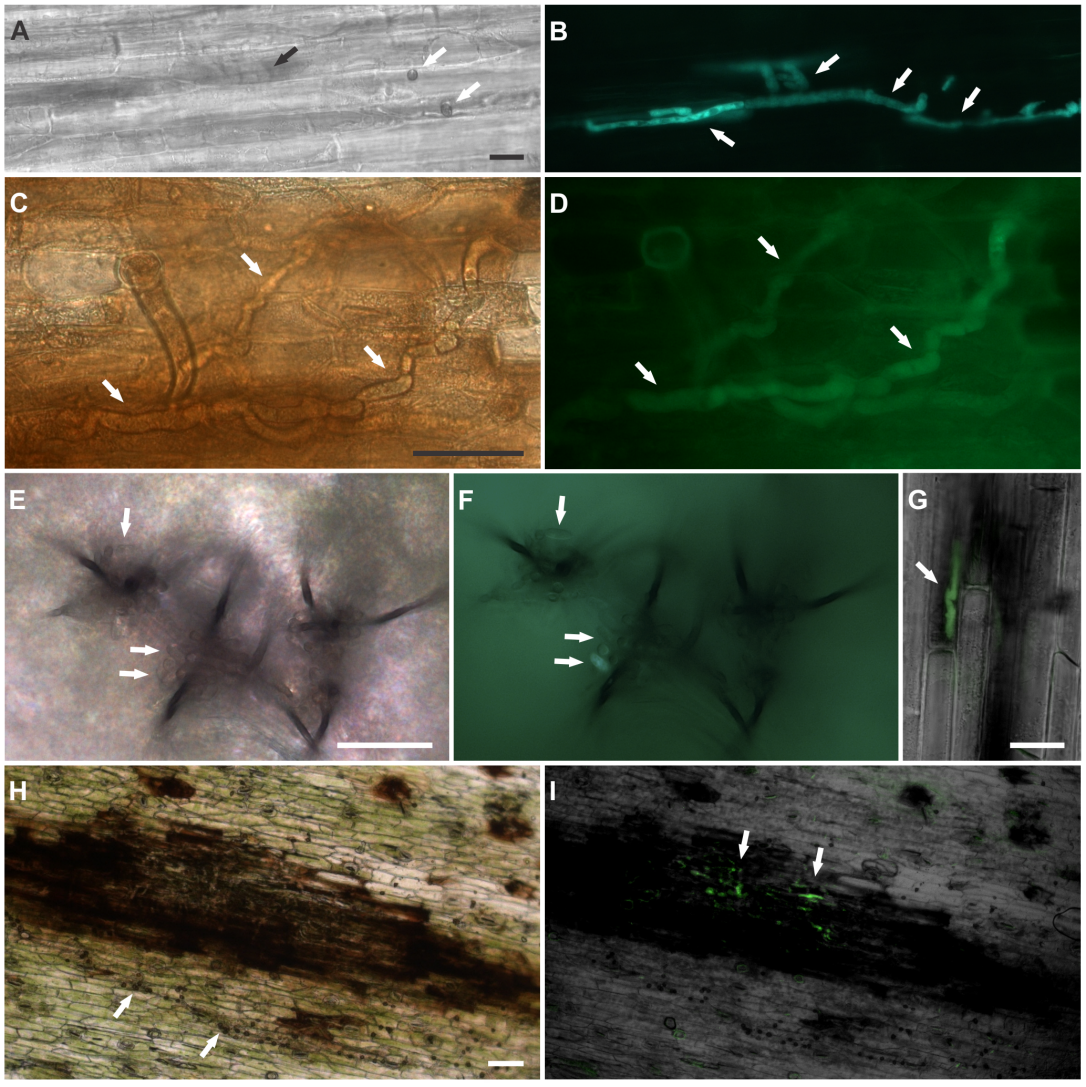
Comparative infection analyses of *C. lindemuthianum* and *Glomerella* strain on tissues of the Pérola susceptible cultivar. A) *C. lindemuthianum* tQB02 transformant strain on a hypocotyl at 72 hpi. White arrows indicate appressoria, and the black arrow indicates the location of primary hyphae. B) Same view as in panel A, imaged with fluorescence microscopy showing primary hyphae expressing GFP (white arrows). Bar: 20 µm. C) *Glomerella* tQB01 transformant strain inoculated on a hypocotyl at a site where cell death had been induced. Thick hyphae were observed inside the dead cells by 48 hpi (white arrows). D) Same view as in panel C, imaged with fluorescence microscopy, showing thick hyphae expressing GFP (white arrows). Bar: 20 µm. E) *Glomerella* tQB01 transformant strain forming acervuli and spores (white arrows) at 72 hpi on a leaf that had tissue been killed by treatment with dry ice. F) Same view, imaged with fluorescence microscopy showing spores (white arrows). Bar: 20 µm. G) Merged image taken with light and fluorescence microscopy showing co inoculation on hypocotyl using transformant teleomorphic strain (tQB01) and wild type anamorphic strain (LV115). tQB01 expressing GFP on hyphae (white arrow) growing on anthracnose lesion at 120 hpi. Bar: 20 µm. H) Co inoculation on hypocotyls at 120 hpi using the transformant *C. lindemuthianum* strain (tQB02) and the perithecial wild type *Glomerella* strain (UFLAG06). Appressoria of the UFLAG06 strain have formed around the lesion (white arrows). I) Same image taken by fluorescence microscopy showing hyphae of the tQB02 strain expressing GFP in the lesion (white arrows). Bar: 20 µm.

The strains UFLAG06 and LV115 were transformed to produce the GFP-expressing transfomants tQB01 and tQB02, respectively. Inoculations with the GFP-expressing strains facilitated observation of post-penetration structures including infection vesicles, and primary and secondary hyphae of *C. lindemuthianum* (tQB02) (Figure 10A and B). Similar structures were never observed in inoculations of living host tissues using strain tQB01, which was derived from the perithecial monoascospore *Glomerella* strain UFLAG06.

When strain tQB01 was inoculated on sites where localized cell death had been induced by treatment with dry ice, thick hyphae were observed inside the dead cells by 48 hpi (Figure 10C and D). Formation of acervuli was observed on the killed inoculated tissues by 72 hpi (Figure 10E and F). The formation of thick hyphae and acervuli was observed equally in the tissues of plants that were either resistant or susceptible to anthracnose (not shown).

#### Co-inoculations

Hyphae of the *Glomerella* strain (tQB01), expressing GFP could be seen growing within anthracnose lesions caused by the *C. lindemuthianum* wild type strain LV115 in co-inoculations (Figure 10G). In samples inoculated with the transformed *C. lindemuthianum* strain (tQB02), hyphae expressing GFP were observed growing in lesions, and there was an intensive formation of appressoria of the *Glomerella* wild type strain UFLAG06 around these lesions (Figure 10H and I).

## Discussion

Bean anthracnose, caused by *C. lindemuthianum*, is a common and economically damaging disease in Brazil. The ability of the pathogen to rapidly overcome sources of host resistance is not well understood, but the frequent recovery of fertile *Glomerella* strains from anthracnose lesions suggested sexual recombination as one possibility. Previous studies have identified *Glomerella* strains recovered from anthracnose lesions as *Glomerella lindemuthiana* or *Glomerella cingulata* f.sp *phaseoli*, assuming them to be the teleomorphs of *C. lindemuthianum* [11], [13], [32], [34]. However, the results of the current study do not support the hypothesis that these *Glomerella* isolates are related to *C. lindemuthianum*. Instead, most of the isolates appear to belong to an unknown *Glomerella* spp. that lives on the bean as an epiphyte, and grows opportunistically in anthracnose lesions caused by *C. lindemuthianum*. A second group of isolates, belonging to the *C. gloeosporioides* species complex, appears to be comprised of weak pathogens of bean that also take advantage of the lesions caused by the more destructive *C. lindemuthianum*.

The *Glomerella* isolates could be distinguished morphologically from a previously characterized population of *C. lindemuthiaum* from common bean [33]. The *Glomerella* colonies grew faster, and their conidia germinated at a higher rate and were smaller than those of *C. lindemuthianum* [33], [49], [50], [51], [52], [53]. Single ascospores recovered from *Glomerella* perithecia gave rise either to fertile strains that produced ascospores and few or no conidia, or to infertile strains that produced only conidia. Both types could be recovered from a single perithecium. The ascospores were larger than the conidia, and they germinated more quickly and at higher rates. Conidial anastomosis tubes (CATs) are a potential mechanism for horizontal transfer of genes and chromosomes, and thus for asexual recombination [32], [54], [55]. Ascospores never formed CATs, but the conidia of a majority of the conidial strains produced them at varying rates (depending on the strain), similar to previous observations reported for *C. lindemuthianum* strains [33]. The ability of *Glomerella* to produce both sexual and asexual progeny may confer the advantages of both: the potential for sexual recombination, and a greater ability of sexual spores to survive and germinate (due to their larger size, and presumably food reserves), combined with the ability of asexual spores to generate CATs for asexual recombination.

All but one of the *Glomerella* isolates produced a septum in the germinating spore. A lack of septum formation during germination is a trait that is considered to be diagnostic for the *C. orbiculare* species complex of *Colletotrichum*, comprised of *C. lindemuthianum*, *C. orbiculare*, *C. trifolii* and *C. malvarum* [53], [56], [57], [58], [59], [60], [61]. The *C. lindemuthianum* LV115 strain used as a control in the current study did not form a septum during germination. However, it should be noted that in another recent study [33], a few pathogenic, presumptive *C. lindemuthianum* strains formed a septum in the germinating conidia, so it may be that this characteristic is not universally definitive of the species.

Molecular data also supported a division between *Glomerella* and *C. lindemuthianum* strains from bean. Separation of a small subset of the strains into different groups by random amplified polymorphic DNA (RAPD) fingerprinting has been reported in earlier studies [5], [62]. In the current study, primers designed to amplify the HMG region of the MAT1-2-1 gene of *C. lindemuthianum* [10] failed to amplify any of the *Glomerella* strains, although they successfully amplified the expected fragment from a large collection of asexual, pathogenic *C. lindemuthianum* strains from the same regions of Brazil [33]. A primer pair specific for the HMG sequence of the *Glomerella* strains was developed by first amplifying the region using degenerate universal primers [42]. The new specific primer pair amplified an HMG fragment from nearly all of the *Glomerella* strains. There were six strains that amplified very poorly with these primers: UFLAG85-1, UFLAG86-1, UFLAG89-1, UFLAG92-1, UFLAG93-1 and UFLAG99-1. These six strains were all conidial, and five of the six were also completely infertile in confrontations with all other *Glomerella* strains. These same six strains were the only ones that sporulated on bean pods, and they were also the only ones that produced symptoms on common bean, albeit very mild symptoms. These six strains (hereafter referred to as *Glomerella* group II) thus represent a population that is distinct both from *C. lindemuthianum*, and from the remainder of the *Glomerella* isolates in this study (hereafter referred to as *Glomerella* group I).

Phylogenetic analysis of the HMG as well as ITS sequences confirmed that the *Glomerella* group 1 strains are unrelated to *C. lindemuthianum*. Their closest affinity was to *C. magna*, a pathogen of cucurbits that appears to be separate from other characterized groups of *Colletotrichum* [63]. *C. magna* has been described as both a pathogen of cucurbits, and as an endophyte in other plant species [64], [65]. The ITS sequences of *Glomerella* group I were identical or similar, based on BLAST analyses, to those of a small number of other strains in Genbank that had been identified as either *C. gloeosporioides* or *G. cingulata*. However, these strains were separate in the ITS phylogram from verified ex-epitype specimens of the *C. gloeosporioides* species complex, and so it is likely that these were misidentified. Most of the isolates (JQ936233, JQ936232, JQ936217, JX258742, JX258680, JX258688, and JX258795) were identified as endophytes recovered from soybean in Brazil [66]. One of the isolates (JQ753986) was identified as an endophyte isolated from common bean leaves in Brazil (Costa, Queiroz, and Gonzaga, unpublished). Most of the rest of the isolates were also identified as plant endophytes.

The *Glomerella* group II isolates fell within the verified *C. gloeosporioides* species complex, based on their ITS sequences. The *C. gloeosporioides* species complex has recently been revised [67]. A multigene phylogeny identified a large number of individual species within this complex. The ITS sequence alone is insufficient for identification of most of these species [67], but we can see that the *Glomerella* group II isolates are distinct from a few of them, including *C. boninense* and *C. kahawe*. Final identification of the group I and II isolates must await a more complete phylogenetic analysis.

Sexual behavior among the *Glomerella* strains from bean was similar in some ways to a series of descriptions of a *G. cingulata* population isolated from morning glory, published in the first half of the 20^th^ century [17]–[28]. These extensive studies resulted in a theory of sexual compatibility in *Glomerella* known as unbalanced heterothallism, where loss of fertility occurs as a result of frequent mutations in the many required steps for full self-fertility, and compatibility is a result of genetic complementation [27]. The recovery of both fertile and infertile progeny strains, with clumped versus scattered arrangements of conidia and perithecia, from individual perithecia of the bean *Glomerella* strains was reminiscent of the morning glory studies. A majority of the recovered progeny were infertile (53 of 73, approximately 75%) suggesting a large propensity to lose fertility. This could be due to the occurrence of a very large number of different mutations, or one or a few common mutations. About half of the infertile strains (28 of 53, 53%) could not be complemented by any of the other strains, suggesting that they shared a common mutation with all of those strains. The strains that were complemented could only be complemented by fertile strains, and not by one another, which also argues in favor of a single common mutation. Most of these strains could be complemented by diffusible substances from certain fertile strains (across membranes). Presence of diffusible hormones inducing self-fertility has been described previously in *Glomerella* [26]. It is possible that these strains are deficient in ability to produce a sexual hormone, and only some of the fertile strains produce a version of the hormone that is recognizable to them. Some strains could be complemented only after contact with certain fertile strains, suggesting complementation by factors acting post-fusion. Interestingly, some strains could be complemented in both ways, by different strains. Three different infertile progeny shared a single pattern of complementation, but otherwise each pattern of complementation was unique. This suggests that each infertile strain may actually have multiple mutations, some common to all, and some unique. All of the fertile strains had different patterns of complementation, suggesting that they don’t all share the same mating-related genes, even though all of the versions apparently function to condition self-fertility. If the unbalanced heterothallism theory can be applied to the *Glomerella* strains from common bean, it suggests that there are a very large number of genes, with a high level of redundancy, that condition compatibility and mating in this fungus. It will be very interesting to explore the mating behavior of this group of strains further, including establishing the existence of recombination in combinations where fertility results only after contact.

Recent studies have revealed other mechanisms that might be involved in the evolution of *mat* loci, and in transitions from heterothallism to homothallism. For example, multiple shifts in the reproductive mode have occurred during the evolutionary history of *Neurospora*, where retrotransposons within *mat* loci have facilitated unequal crossovers or translocation, resulting in relocation of genes of both mating types into the same haploid genome [68]. Transposable elements have been observed within the *mat* loci of numerous Ascomycetes and Basidiomycetes [69]–[72].

The *Glomerella* strains are much less aggressive to common bean than *C. lindemuthianum*. As reported in an earlier preliminary study [32], the *Glomerella* spores germinated with an extremely high efficiency on bean plants within 24 hours, producing masses of melanized appressoria and superficial hyphae. These structures resulted in visible brown flecks at the sites of inoculation, which may explain the mild symptoms that were previously reported to result from inoculation with these strains [5], [11], [13], [32]. In the current study, post-infection processes of a representative of the Type I *Glomerella* strains including penetration and colonization of plant tissues, were compared with *C. lindemuthianum* in detail. These studies revealed that this *Glomerella* strain was completely unable to penetrate or internally colonize living common bean tissues. However, it produced abundant appressoria and epiphytic mycelium that grew along the anticlinal walls of the epidermal cells. For this reason, we do not suggest that these fungi are “endophytes” in the commonly understood sense of occupying the interior of the host tissues. There were no differences in the behavior of the perithecial versus conidial sibling strains. The anthracnose-resistant common bean cultivar significantly reduced the ability of *C. lindemuthianum* to germinate, form appressoria, and colonize the tissue. In contrast, there was no effect of host resistance to anthracnose on the *Glomerella* strains, which germinated and formed appressoria and superficial hyphae at the same rate on resistant versus susceptible cultivars. When localized injury of the common bean leaf tissue was caused by contact with dry ice, the *Glomerella* strains produced thick hyphae that rapidly colonized the dead cells, and eventually gave rise to acervuli and setae in both susceptible and resistant cultivars. The co-infection experiments demonstrated that *C. lindemuthianum* anthracnose lesions can be colonized by *Glomerella* strains. Thus, in the field, we propose that these *Glomerella* strains exist as epiphytes on the surface of the bean tissues, and opportunistically colonize and sporulate within anthracnose lesions caused by *C. lindemuthianum*, explaining the recovery of both organisms from the same lesions.

*Colletotrichum* (and its teleomorph *Glomerella*) is a large genus with diverse lifestyles ranging from necrotrophic pathogenic to latent and epiphytic [73]. *Colletotrichum* is a common epiphytic inhabitant of foliar tissues, where it can persist as spores, appressoria, or mycelium [74]–[78]. The relationship between pathogenicity, endophytism, epiphytism, and saprophytism is not clear in *Colletotrichum* although it appears that some species can exhibit more than one lifestyle in their life cycle, e.g. latent epiphytes or endophytes can transform to pathogens or saprophytes as host status changes (tissue senescence or necrosis) [65], [67], [76], [79], [80]. Common bean in Brazil is cultivated across three seasons each year under widely varying environmental conditions, resulting in a complex biosystem and the opportunity for rapid evolution of new common bean-associated species. The specific nature of the relationship between *C. lindemuthianum* and the *Glomerella* strains on common bean remains to be determined, however the frequency of their association suggests that there may be an inter-dependence of the two species. The *Glomerella* may rely on the *C. lindemuthianum* to produce dead tissues on which it can sporulate. Formation of appressoria by *Colletotrichum* is known to trigger defenses in host species [81], so it will be important to determine whether co-inoculation with *Glomerella*, apparently an aggressive colonizer of the phylloplane, can “prime” defense mechanisms in common bean, perhaps increasing its resistance to *C. lindemuthianum*. The epiphytic *Glomerella* mycelium may compete for phylloplane resources, or they may produce inhibitory antibiotics, affecting the survival and germination of the *C. lindemuthianum* spores [78]. The *Glomerella* strains may have significant potential as biocontrol organisms. Furthermore, given that both organisms are able to form CATS, and that it has been shown previously that CATs can form between members of different species of *Colletotrichum* [82], there is at least the potential for genetic exchange between the two organisms, something that could add to the potential diversity of the *C. lindemuthianum*. This is especially true when we consider how extremely variable the population of *Glomerella* strains on common bean leaves is, possibly related to their ability to undergo sexual recombination. The recognition of these interdependent *Colletotrichum* communities associated with common bean anthracnose adds a new dimension to our understanding of the nature and potential mechanisms of phenotypic variation and adaptability of this important pathogen.

## Supporting Information

Table S1 **Host cultivar from which the infected tissues were collected, origin and type of the strains used in this study.** * Strains used in infection assays. **transformant strains expressing green fluorescent protein and hygromicin resistance used in infection assays. Strains in bold were used for ITS sequencing; strains in italics were used for HMG sequencing. Strains in bold italics were used for both. Underlined strains were used in morphological characterization experiments. NA = not applicable; a = common bean lines from breeding programs that do not have a commercial name yet; b = single-spored field isolates; c = not applicable, transformed strains obtained in the laboratory; d = *C. lindemuthianum* strains are anamorphic, and produce only conidia.
(DOCX)

Table S2 **Fertile confrontations among conidial and perithecial strains.** ^CA^ = conidial A strains; ^CB^ = conidial B strains;^+^ =  perithecial plus strains; ^−^ = perithecial minus strains; I = induced homothalism; confrontations in which lines of fertile perithecia were formed in both the presence and absence of dialysis membrane; L = likely heterothallic; confrontations in which lines of fertile perithecia were formed only in the absence of dialysis membrane.
(DOCX)

File S1 **ITS sequence alignment.**
(TXT)
