# Correction: Characterization of *Glomerella* Strains Recovered from Anthracnose Lesions on Common Bean Plants in Brazil

**DOI:** 10.1371/journal.pone.0100438

**Published:** 2014-06-13

**Authors:** The PLOS ONE Staff

The following information is missing from the Funding section: The information reported in this paper (No. 14-12-005) is part of a project of the Kentucky Agricultural Experiment Station and is published with the approval of the Director.
