# Supplementary material for: Characterization of Glomerella Strains Recovered from Anthracnose Lesions on Common Bean Plants in Brazil
Source: PLoS One. 2014 Mar 14;9(3):e90910. doi: 10.1371/journal.pone.0090910 (PMC3954623; doi:10.1371/journal.pone.0090910)
Supplement: Table S1 — Host cultivar from which the infected tissues were collected, origin and type of the strains used in this study. * Strains used in infection assays. **transformant strains expressing green fluorescent protein and hygromicin resistance used in infection assays. Strains in bold were used for ITS sequencing; strains in italics were used for HMG sequencing. Strains in bold italics were used for both. Underlined strains were used in morphological characterization experiments. NA = not applicable; a = common bean lines from breeding programs that do not have a commercial name yet; b = single-spored field isolates; c = not applicable, transformed strains obtained in the laboratory; d = C. lindemuthianum strains are anamorphic, and produce only conidia. (DOCX) [file pone.0090910.s001.docx]

**Table S1. Host cultivar from which the infected tissues were collected, origin and type of the strains used in this study.**

| **Strain** | **Host Cultivar** | **Location** | **Progenitor** | **Colony Type** | **Conidial**  **Production** | **Strain** | **Host Cultivar** | **Location** | **Progenitor** | **Colony Type** | **Conidial**  **Production** |
| --- | --- | --- | --- | --- | --- | --- | --- | --- | --- | --- | --- |
| *UFLAG01* | Majestoso | Guarapuava | NA^b^ | Plus | none | UFLAG26-1 | Magnífico | Lambari | UFLAG26 | Conidial A | M3 |
| **UFLAG02** | Majestoso | Lavras | NA^b^ | Plus | none | UFLAG29-1 | NA^a^ | Lambari | UFLAG29 | Conidial B | M3 |
| ***UFLAG03-1*** | Majestoso | R. Vermelho | UFLAG03 | Conidial B | M3 | UFLAG30-1 | Magnífico | Lambari | UFLAG30 | Plus | none |
| **UFLAG04** | Majestoso | R.Vermelho | NA^b^ | Plus | none | UFLAG30-2 | Magnífico | Lambari | UFLAG30 | Conidial B | M3 |
| UFLAG05-1 | Majestoso | R. Vermelho | UFLAG05 | Conidial A | M3 | UFLAG34-1 | Magnífico | Lambari | UFLAG34 | Conidial B | M3 |
| **UFLAG05-2** | Majestoso | R. Vermelho | UFLAG05 | Conidial A | M3 | UFLAG35-1 | Pérola | Lambari | UFLAG35 | Conidial B | M3 |
| ***UFLAG06**** | Majestoso | R. Vermelho | NA^b^ | Plus | none | UFLAG36-1 | Pérola | Lambari | UFLAG36 | Conidial B | M3 |
| UFLAG06-1* | Majestoso | R. Vermelho | UFLAG06 | Plus | none | UFLAG37-1 | Pérola | Lambari | UFLAG37 | Conidial B | M3 |
| UFLAG06-2* | Majestoso | R. Vermelho | UFLAG06 | Conidial B | M3 | UFLAG39-1 | NA^a^ | Lambari | UFLAG39 | Minus | none |
| ***UFLAG06-3*** | Majestoso | R. Vermelho | UFLAG06 | Conidial B | M3 | UFLAG41-1 | NA^a^ | Lambari | UFLAG41 | Conidial B | M3 |
| UFLAG07-1 | Majestoso | Lavras | UFLAG07 | Plus | none | UFLAG43-1 | Pérola | Lambari | UFLAG43 | Minus | none |
| UFLAG07-2 | Majestoso | Lavras | UFLAG07 | Conidial A | M3 | UFLAG43-2 | Pérola | Lambari | UFLAG43 | Plus | none |
| **UFLAG07-3** | Majestoso | Lavras | UFLAG07 | Conidial A | M3 | UFLAG45-1 | Majestoso | Lambari | UFLAG45 | Conidial B | M3 |
| ***UFLAG08**** | Majestoso | Lavras | NA^b^ | Minus | none | **UFLAG46-1** | Majestoso | Lambari | UFLAG46 | Conidial A | M3 |
| UFLAG08-1 | Majestoso | Lavras | UFLAG08 | Minus | none | UFLAG47-1 | Majestoso | Lambari | UFLAG47 | Conidial B | M3 |
| UFLAG08-2* | Majestoso | Lavras | UFLAG08 | Minus | none | UFLAG47-2 | Majestoso | Lambari | UFLAG47 | Minus | none |
| UFLAG08-3* | Majestoso | Lavras | UFLAG08 | Conidial A | M3 | UFLAG48-1 | Majestoso | Lambari | UFLAG48 | Conidial B | M3 |
| ***UFLAG08-4*** | Majestoso | Lavras | UFLAG08 | Conidial A | M3 | UFLAG49-1 | Majestoso | Lambari | UFLAG49 | Conidial B | M3 |
| UFLAG10-1 | NA^a^ | Lambari | UFLAG10 | Conidial B | M3 | UFLAG54-1 | NA^a^ | Lambari | UFLAG54 | Conidial A | M3 |
| UFLAG13-1 | Majestoso | Lambari | UFLAG13 | Conidial B | M3 | UFLAG54-2 | NA^a^ | Lambari | UFLAG54 | Plus | none |
| UFLAG15-1 | Majestoso | Lambari | UFLAG15 | Plus | none | UFLAG55-1 | Magnífico | Lambari | UFLAG55 | Conidial B | M3 |
| UFLAG15-2 | Majestoso | Lambari | UFLAG15 | Conidial A | M3 | UFLAG60-1 | NA^a^ | Lambari | UFLAG60 | Conidial B | M3 |
| UFLAG20-1 | Magnífico | Lambari | UFLAG20 | Conidial B | M3 | UFLAG61-1 | NA^a^ | Lambari | UFLAG61 | Conidial B | M3 |
| UFLAG21-1 | Pérola | Lambari | UFLAG21 | Conidial A | M3 | UFLAG64-1 | Magnífico | Lambari | UFLAG64 | Conidial A | M3 |
| UFLAG21-2 | Pérola | Lambari | UFLAG21 | Plus | none | UFLAG68-1 | Pérola | Lavras | UFLAG68 | Conidial A | M3 |
| UFLAG23-1 | Pérola | Lambari | UFLAG23 | Conidial B | M3 | UFLAG73-1 | NA^a^ | Lambari | UFLAG73 | Plus | none |
| UFLAG25-1 | Magnífico | Lambari | UFLAG25 | Conidial B | M3 | UFLAG73-2 | NA^a^ | Lambari | UFLAG73 | Minus | none |

Cont…

| **Strain** | **Host Cultivar** | **Location** | **Progenitor** | **Colony Type** | **Conidial**  **Production** | **Strain** | **Host Cultivar** | **Location** | **Progenitor** | **Colony Type** | **Conidial**  **Production** |
| --- | --- | --- | --- | --- | --- | --- | --- | --- | --- | --- | --- |
| UFLAG74-1 | NA^a^ | Lambari | UFLAG74 | Conidial A | M3 | UFLAG106-2 | Majestoso | Lavras | UFLAG106 | Conidial B | M3 |
| UFLAG75-1 | NA^a^ | Lambari | UFLAG75 | Conidial A | M3 | UFLAG107-1 | Majestoso | Lavras | UFLAG107 | Conidial A | M3 |
| UFLAG79-1 | NA^a^ | Lambari | UFLAG79 | Conidial B | M3 | UFLAG108-1 | Majestoso | Lavras | UFLAG108 | Conidial A | M3 |
| UFLAG82-1 | Majestoso | Lavras | UFLAG82 | Conidial B | M3 | UFLAG109-1 | Majestoso | Lavras | UFLAG109 | Conidial B | M3 |
| UFLAG83-1 | Majestoso | Lavras | UFLAG83 | Conidial B | M3 | UFLAG110-1 | Majestoso | Lavras | UFLAG110 | Conidial B | M3 |
| UFLAG84-1 | Majestoso | Lavras | UFLAG84 | Plus | none | UFLAG111-1 | Majestoso | Lavras | UFLAG111 | Conidial B | M3 |
| **UFLAG85-1** | Majestoso | Lavras | UFLAG85 | Conidial A | Bean Pod | **UFLAG112** | Majestoso | Lavras | NA^b^ | Minus | none |
| UFLAG86-1 | Majestoso | Lavras | UFLAG86 | Conidial A | Bean Pod | UFLAG112-1 | Majestoso | Lavras | UFLAG112 | Minus | none |
| UFLAG88-1 | Majestoso | Lavras | UFLAG88 | Conidial A | M3 | UFLAG113-1 | Majestoso | Lavras | UFLAG113 | Minus | none |
| UFLAG89-1 | Majestoso | Lavras | UFLAG89 | Conidial A | Bean Pod | UFLAG114-1 | Majestoso | Lavras | UFLAG114 | Conidial B | M3 |
| UFLAG91-1 | Majestoso | Lavras | UFLAG91 | Conidial A | M3 | UFLAG116-1 | Majestoso | Lavras | UFLAG116 | Conidial A | M3 |
| UFLAG92-1 | Majestoso | Lavras | UFLAG92 | Conidial B | Bean pod | UFLAG117-1 | Majestoso | Lavras | UFLAG117 | Conidial B | M3 |
| **UFLAG93-1** | Majestoso | Lavras | UFLAG93 | Conidial A | Bean pod | UFLAG118-1 | Majestoso | Lavras | UFLAG118 | Plus | none |
| UFLAG97-1 | Majestoso | Lavras | UFLAG97 | Conidial A | M3 | UFLAG119-1 | Majestoso | Lavras | UFLAG119 | Minus | none |
| UFLAG98-1 | Majestoso | Lavras | UFLAG98 | Conidial A | M3 | ***LV115**** | Carioca | Patos de Minas | NA^b^ | Conidial^d^ | Bean pod |
| **UFLAG99-1** | Majestoso | Lavras | UFLAG99 | Conidial A | Bean pod | ***LV117*** | NA^a^ | Lavras | NA^b^ | Conidial^d^ | Bean pod |
| UFLAG101-1 | Majestoso | Lavras | UFLAG101 | Plus | none | ***LV120*** | Talismã | Lambari | NA^b^ | Conidial^d^ | Bean pod |
| UFLAG104-1 | Majestoso | Lavras | UFLAG104 | Minus | none | *CLKY1* | Kentucky Wonder | Lexington (EUA) | NA^b^ | Conidial^d^ | Bean pod |
| UFLAG104-2 | Majestoso | Lavras | UFLAG104 | Conidial A | M3 | tQB01** hph, sgfp | NA^c^ | NA^c^ | UFLAG06 | Plus | none |
| UFLAG106-1 | Majestoso | Lavras | UFLAG106 | Plus | none | tQB02** hph, sgfp | NA^c^ | NA^c^ | LV115 | Conidial^d^ | M3 |

* Strains used in infection assays. **transformant strains expressing green fluorescent protein and hygromicin resistance used in infection assays.

Strains in bold were used for ITS sequencing; strains in italics were used for HMG sequencing. Strains in bold italics were used for both. Underlined strains were used in morphological characterization experiments. NA = not applicable; a = common bean lines from breeding programs that do not have a commercial name yet; b = single-spored field isolates; c = not applicable, transformed strains obtained in the laboratory; d = *C.lindemuthianum* strains are anamorphic, and produce only conidia.
