# Supplementary material for: Characterization of Glomerella Strains Recovered from Anthracnose Lesions on Common Bean Plants in Brazil
Source: PLoS One. 2014 Mar 14;9(3):e90910. doi: 10.1371/journal.pone.0090910 (PMC3954623; doi:10.1371/journal.pone.0090910)
Supplement: Table S2 — Fertile confrontations among conidial and perithecial strains. CA = conidial A strains; CB = conidial B strains;+ = perithecial plus strains; − = perithecial minus strains; I = induced homothalism; confrontations in which lines of fertile perithecia were formed in both the presence and absence of dialysis membrane; L = likely heterothallic; confrontations in which lines of fertile perithecia were formed only in the absence of dialysis membrane. (DOCX) [file pone.0090910.s002.docx]

**Table S2. Fertile confrontations among conidial and perithecial strains.**

| Strains | UFLAG07-1^+^ | UFLAG15-1^+^ | UFLAG21-2^+^ | UFLAG30-1^+^ | UFLAG43-2^+^ | UFLAG54-2^+^ | UFLAG73-1^+^ | UFLAG84-1^+^ | UFLAG101-1^+^ | UFLAG106-1^+^ |
| --- | --- | --- | --- | --- | --- | --- | --- | --- | --- | --- |
| UFLAG05-1^CA^ |  |  |  |  |  |  |  |  |  |  |
| UFLAG07-2^CA^ |  |  |  |  |  |  |  |  |  |  |
| UFLAG15-2^CA^ |  | L |  |  |  |  | I |  |  |  |
| UFLAG21-1^CA^ |  |  | L |  |  |  |  |  |  |  |
| UFLAG26-1^CA^ |  |  |  |  |  |  |  |  |  |  |
| UFLAG46-1^CA^ |  |  |  |  |  |  |  |  |  |  |
| UFLAG54-1^CA^ |  |  | I |  |  | L |  |  |  |  |
| UFLAG64-1^CA^ |  |  |  |  |  |  |  |  |  |  |
| UFLAG68-1^CA^ |  |  |  |  |  |  |  |  |  |  |
| UFLAG74-1^CA^ |  |  |  |  |  |  |  | I |  |  |
| UFLAG75-1^CA^ |  |  |  |  |  |  |  |  |  |  |
| UFLAG85-1^CA^ |  |  |  |  |  |  |  |  |  |  |
| UFLAG86-1^CA^ |  |  |  |  | I |  |  |  |  |  |
| UFLAG88-1^CA^ |  |  |  |  |  |  |  |  |  |  |
| UFLAG89-1^CA^ |  |  |  |  |  |  |  |  |  |  |
| UFLAG91-1^CA^ |  |  |  |  |  |  |  |  |  |  |
| UFLAG93-1^CA^ |  |  |  |  |  |  |  |  |  |  |
| UFLAG97-1^CA^ |  |  |  |  |  |  |  |  |  |  |
| UFLAG98-1^CA^ |  |  |  |  |  |  |  |  | L |  |
| UFLAG99-1^CA^ |  |  |  |  |  |  |  |  |  |  |
| UFLAG104-2^CA^ |  |  | I |  |  |  |  |  | L |  |
| UFLAG107-1^CA^ |  |  |  |  |  |  |  |  |  |  |
| UFLAG108-1^CA^ |  |  |  |  |  |  |  |  | L |  |
| UFLAG116-1^CA^ |  |  |  |  |  |  |  |  |  |  |

Cont…

| Strains | UFLAG118-1^+^ | UFLAG08-1^-^ | UFLAG39-1^-^ | UFLAG43-1^-^ | UFLAG47-2^-^ | UFLAG73-2^-^ | UFLAG104-1^-^ | UFLAG112-1^-^ | UFLAG113-1^-^ | UFLAG119-1^-^ |
| --- | --- | --- | --- | --- | --- | --- | --- | --- | --- | --- |
| UFLAG05-1^CA^ |  |  |  |  |  |  |  |  |  |  |
| UFLAG07-2^CA^ |  | I | I |  |  |  |  |  |  |  |
| UFLAG15-2^CA^ |  |  |  |  |  |  |  |  |  |  |
| UFLAG21-1^CA^ |  |  |  |  |  |  |  |  |  |  |
| UFLAG26-1^CA^ |  |  |  |  |  |  |  |  |  | I |
| UFLAG46-1^CA^ |  |  |  |  |  |  |  |  |  | I |
| UFLAG54-1^CA^ |  |  |  |  |  |  |  |  |  |  |
| UFLAG64-1^CA^ |  |  |  |  |  |  |  |  |  |  |
| UFLAG68-1^CA^ |  |  |  |  |  |  |  |  |  |  |
| UFLAG74-1^CA^ |  |  |  |  |  |  |  |  |  | I |
| UFLAG75-1^CA^ | L | I |  |  |  |  |  | L | L |  |
| UFLAG85-1^CA^ |  |  |  |  |  |  |  |  |  |  |
| UFLAG86-1^CA^ |  |  |  | I |  |  |  |  |  | I |
| UFLAG88-1^CA^ |  |  |  |  |  |  |  |  |  |  |
| UFLAG89-1^CA^ |  |  |  |  |  |  |  |  |  |  |
| UFLAG91-1^CA^ |  |  |  |  |  |  |  |  |  |  |
| UFLAG93-1^CA^ |  |  |  |  |  |  |  |  |  |  |
| UFLAG97-1^CA^ |  |  |  |  |  |  |  |  |  |  |
| UFLAG98-1^CA^ |  |  |  |  |  |  |  |  |  |  |
| UFLAG99-1^CA^ |  |  |  |  |  |  |  |  |  |  |
| UFLAG104-2^CA^ |  |  |  |  |  |  | L |  |  |  |
| UFLAG107-1^CA^ |  |  |  | I |  |  |  |  |  |  |
| UFLAG108-1^CA^ |  |  |  |  |  |  | L |  |  |  |
| UFLAG116-1^CA^ |  |  |  |  |  |  |  |  | L |  |

Cont…

| Strains | UFLAG07-1^+^ | UFLAG15-1^+^ | UFLAG21-2^+^ | UFLAG30-1^+^ | UFLAG43-2^+^ | UFLAG54-2^+^ | UFLAG73-1^+^ | UFLAG84-1^+^ | UFLAG101-1^+^ | UFLAG106-1^+^ |
| --- | --- | --- | --- | --- | --- | --- | --- | --- | --- | --- |
| UFLAG10-1^CB^ |  |  |  |  |  |  |  |  |  |  |
| UFLAG13-1^CB^ |  |  |  |  |  |  |  |  |  |  |
| UFLAG20-1^CB^ |  |  |  |  |  |  |  |  |  |  |
| UFLAG23-1^CB^ |  |  |  |  |  |  |  |  |  |  |
| UFLAG25-1^CB^ |  |  |  |  |  |  |  |  |  |  |
| UFLAG29-1^CB^ |  |  |  |  |  |  |  |  |  |  |
| UFLAG30-2^CB^ |  |  |  |  |  |  |  |  |  |  |
| UFLAG34-1^CB^ |  |  |  |  |  |  |  |  |  |  |
| UFLAG35-1^CB^ |  |  |  |  |  |  |  |  |  |  |
| UFLAG36-1^CB^ |  |  |  |  |  |  |  |  |  |  |
| UFLAG37-1^CB^ |  |  |  |  |  |  |  |  |  |  |
| UFLAG41-1^CB^ |  |  |  |  |  |  |  |  |  |  |
| UFLAG45-1^CB^ |  | I |  |  |  |  |  |  |  |  |
| UFLAG47-1^CB^ |  | L |  |  | L |  |  |  |  |  |
| UFLAG48-1^CB^ |  |  |  |  |  |  |  |  |  |  |
| UFLAG49-1^CB^ |  |  |  |  |  |  |  |  | L |  |
| UFLAG55-1^CB^ |  |  |  |  |  | I |  |  |  |  |
| UFLAG60-1^CB^ |  |  |  |  |  |  |  |  |  |  |
| UFLAG61-1^CB^ |  |  |  |  |  |  |  |  |  |  |
| UFLAG79-1^CB^ |  |  |  |  | I |  |  | I |  |  |
| UFLAG82-1^CB^ |  |  |  |  |  |  |  |  |  |  |
| UFLAG83-1^CB^ |  |  |  |  |  |  |  | I |  |  |
| UFLAG92-1^CB^ |  |  |  |  |  |  |  |  |  |  |
| UFLAG106-2^CB^ |  |  |  |  |  |  |  |  |  | L |

Cont…

| Strains | UFLAG118-1^+^ | UFLAG08-1^-^ | UFLAG39-1^-^ | UFLAG43-1^-^ | UFLAG47-2^-^ | UFLAG73-2^-^ | UFLAG104-1^-^ | UFLAG112-1^-^ | UFLAG113-1^-^ | UFLAG119-1^-^ |
| --- | --- | --- | --- | --- | --- | --- | --- | --- | --- | --- |
| UFLAG10-1^CB^ |  |  |  |  |  |  |  |  |  |  |
| UFLAG13-1^CB^ |  |  |  |  |  |  |  |  |  |  |
| UFLAG20-1^CB^ |  |  |  |  |  |  |  |  |  |  |
| UFLAG23-1^CB^ |  |  |  |  |  |  |  |  |  |  |
| UFLAG25-1^CB^ |  |  |  |  |  |  |  |  |  |  |
| UFLAG29-1^CB^ |  |  |  |  |  |  |  |  |  |  |
| UFLAG30-2^CB^ |  |  |  |  |  |  |  |  |  |  |
| UFLAG34-1^CB^ |  |  |  |  |  |  |  |  |  |  |
| UFLAG35-1^CB^ |  |  |  |  |  |  |  |  |  |  |
| UFLAG36-1^CB^ |  |  |  |  |  |  |  |  |  |  |
| UFLAG37-1^CB^ |  |  |  |  |  |  |  |  |  |  |
| UFLAG41-1^CB^ |  |  |  |  |  |  |  |  |  |  |
| UFLAG45-1^CB^ |  |  |  |  | L |  |  |  |  |  |
| UFLAG47-1^CB^ |  |  |  |  | L |  |  |  |  |  |
| UFLAG48-1^CB^ |  |  |  |  |  |  |  |  |  |  |
| UFLAG49-1^CB^ |  |  |  |  |  |  | L |  |  |  |
| UFLAG55-1^CB^ |  |  |  |  |  |  |  |  |  |  |
| UFLAG60-1^CB^ |  |  |  |  |  |  |  |  |  |  |
| UFLAG61-1^CB^ |  |  |  |  |  |  |  |  |  |  |
| UFLAG79-1^CB^ |  | I |  | I |  |  |  |  |  |  |
| UFLAG82-1^CB^ |  |  |  |  |  |  |  |  |  |  |
| UFLAG83-1^CB^ |  |  |  |  |  |  |  |  |  |  |
| UFLAG92-1^CB^ |  |  |  |  |  |  |  |  |  |  |
| UFLAG106-2^CB^ |  |  |  |  |  |  |  |  |  |  |

Cont…

| Strains | UFLAG07-1^+^ | UFLAG15-1^+^ | UFLAG21-2^+^ | UFLAG30-1^+^ | UFLAG43-2^+^ | UFLAG54-2^+^ | UFLAG73-1^+^ | UFLAG84-1^+^ | UFLAG101-1^+^ | UFLAG106-1^+^ |
| --- | --- | --- | --- | --- | --- | --- | --- | --- | --- | --- |
| UFLAG109-1^CB^ |  |  |  |  |  |  |  |  |  |  |
| UFLAG110-1^CB^ |  |  |  |  |  |  |  |  | L |  |
| UFLAG111-1^CB^ |  |  |  | L |  |  |  |  |  |  |
| UFLAG114-1^CB^ |  |  |  |  |  |  |  |  |  |  |
| UFLAG117-1^CB^ |  |  |  |  |  |  |  |  | L |  |

| Strains | UFLAG118-1^+^ | UFLAG08-1^-^ | UFLAG39-1^-^ | UFLAG43-1^-^ | UFLAG47-2^-^ | UFLAG73-2^-^ | UFLAG104-1^-^ | UFLAG112-1^-^ | UFLAG113-1^-^ | UFLAG119-1^-^ |
| --- | --- | --- | --- | --- | --- | --- | --- | --- | --- | --- |
| UFLAG109-1^CB^ |  |  |  |  |  |  |  |  |  |  |
| UFLAG110-1^CB^ |  |  |  |  |  |  | L |  |  |  |
| UFLAG111-1^CB^ |  |  |  |  |  |  |  | L |  |  |
| UFLAG114-1^CB^ |  |  |  |  |  |  |  | I |  | I |
| UFLAG117-1^CB^ | L |  |  |  |  |  | L |  |  |  |

^CA^ = conidial A strains; ^CB^ = conidial B strains; ^+^ = perithecial plus strains; ^-^ = perithecial minus strains; I = induced homothalism; confrontations in which lines of fertile perithecia were formed in both the presence and absence of dialysis membrane; L = likely heterothallic; confrontations in which lines of fertile perithecia were formed only in the absence of dialysis membrane.
